# Differences in risky sexual behaviors and HIV prevalence of circumcised and uncircumcised men in Uganda: evidence from a 2011 cross-sectional national survey

**DOI:** 10.1186/1742-4755-11-25

**Published:** 2014-03-24

**Authors:** Simon PS Kibira, Elizabeth Nansubuga, Nazarius M Tumwesigye, Lynn M Atuyambe, Fredrick Makumbi

**Affiliations:** 1 School of Public Health, Makerere University, Kampala, Uganda; 2 School of Statistics and Planning, Makerere University, Kampala, Uganda

**Keywords:** Safe male circumcision, Risky sexual behavior, HIV, Men, Uganda

## Abstract

**Background:**

Safe male circumcision (SMC) is a known efficacious intervention in the prevention of heterosexual HIV acquisition. However, there are perceptions that SMC may lead to behavior disinhibition towards risky sexual behaviors. We assessed the association between male circumcision, risky sexual behaviors and HIV prevalence among men in a nationally representative sample.

**Methods:**

Data was extracted from the Uganda AIDS Indicator Survey (2011), a stratified two-stage cluster sample, with a total of 7,969 ever sexually active men aged 15–59 years. The association between risky sexual behaviors (*non- marital/non-cohabiting sexual relations, non-use of condoms, transactional sex, multiple (4+) lifetime partners*) and male circumcision status were determined using odds ratios (OR) and their 95% confidence intervals, through logistic regression models. All analyses were conducted in Stata version 12.

**Results:**

Overall, the prevalence of male circumcision was 28%; higher among men aged 25–34 years, 32%, and lowest among those aged 45–59 years, 18%. HIV prevalence was significantly lower among the circumcised, 4.8% compared to the uncircumcised men, 7.8% (p < 0.001). The commonest risky sexual behaviors were multiple life-time sexual partners (4+), 59%; non-use of condoms with non-marital sexual partners, 55%; and having non-marital sex, 33%. In comparison with the uncircumcised, circumcised men had higher odds of engaging in non-marital sex AOR = 1.26 (95% CI: 1.05-1.52), reporting multiple (4+) life-time partners, AOR = 1.46 (95% CI: 1.27-1.67). The odds of non-use of condoms with a non-marital partner were also significantly lower among the circumcised compared to the uncircumcised men, AOR = 0.79 (95% CI: 0.63-0.98).

**Conclusions:**

Although risky sexual behaviors were more common among circumcised men, HIV prevalence was lower among the circumcised men relative to the uncircumcised. These observations suggest a need to promote the already known HIV intervention strategies especially among the circumcised men.

## Background

Male circumcision is the surgical removal of the foreskin of the human penis. Foreskin is one of the risk factors for Human Immunodeficiency Virus (HIV) acquisition from infected women to men [1]. Circumcision is practiced for religious, cultural, social as well as medical reasons in various settings [2]. The three randomized clinical trials (RCTs) of male circumcision conducted in sub-Saharan Africa; Uganda [3], Kenya [4] and South Africa [5], showed an average of 60% reduction in HIV-acquisition. Other benefits of medical male circumcision included a lower risk of sexually transmitted infections (STIs) such as female genital ulcerations, bacterial vaginosis, trichomoniasis, human papilloma virus and chlamydia in female partners [3,6-8]. Based on these study findings, the World Health Organization and Joint United Nations Program on HIV/AIDS in 2007 recommended the adoption of male circumcision as part of the comprehensive strategy to reduce heterosexually acquired HIV infection in countries with high HIV prevalence and lower levels of male circumcision [9].

Although male circumcision was primarily practiced for religious and cultural reasons in Uganda, a safe male circumcision (SMC) policy was launched in 2010 as part of the comprehensive strategy on HIV prevention to complement the “ABC” strategy (abstinence, being faithful to one partner and condom use). The goal of the SMC policy is to contribute to the reduction of HIV and other STIs [2], and to establish a research agenda focusing on male circumcision services towards HIV prevention as one of the key objectives. This policy also recommends the integration of SMC services in the HIV prevention and sexual and reproductive health care services, while targeting all males including neonates whose parents and/or guardians consent to the procedure.

As a result of the policy, several strategies including limited offer of free circumcision at public health facilities, mobilization and sensitization of the population have been put in place to scale-up SMC in Uganda. Furthermore, in light of the research agenda of the policy, studies [10,11] have also been undertaken to provide evidence based information useful in future programming of circumcision programs or services in the country.

Although Uganda recorded a slight increase in the percentage of circumcised adult males aged 15 – 59 years from 25% in 2004 to 27% in 2011, the overall adult HIV prevalence increased from 6.4% to 7.3%, and from 5.4% to 6.1% in adult men in the same period [12,13]. This increase has been associated with improved survival of HIV-infected persons who are enrolled in HIV-care and treatment [12], and the increase in risky sexual behavior (RSB) defined as concurrent multiple partnerships, non-consistent condom use with non-marital and non-cohabiting partners, use of alcohol just before sex and transactional sex .

Increases in RSB may be explained by behavior risk compensation where people tend to adjust their behavior in response to the perceived level of risk. Usually people behave less cautiously when they feel more protected and become more cautious when they feel a higher level of risk. Because SMC is promoted as one of the prevention methods for HIV, there is a possibility of some people perceiving it as very highly protective [14] against HIV infection. In this context, it is possible that negative sexual behavior changes [15-17] may result among circumcised men [18,19].

Although the increase in HIV prevalence has been partly explained by improved survival of HIV+ persons in care, anecdotal reports suggest an increase in RSB. However, the role of SMC on the increase in RSB is not clearly known, even as SMC policy is implemented in Uganda. We therefore compare sexual behaviors and HIV prevalence between circumcised and non-circumcised men from a nationally representative sample in Uganda.

## Methods

Data are drawn from the 2011 Uganda AIDS Indicator Survey (AIS), a nationally representative sample obtained from a stratified two-stage cluster sampling strategy [12]. Clusters were selected from each stratum at the first stage, while the second stage involved selecting households for interview to obtain eligible respondents. The strata were defined as urban/rural and sub-regions while the clusters were enumeration areas as of the 2010 Uganda National Household Survey updates [12]. Data for this survey were collected between February and September 2010, led by the Ministry of Health working with ICF international, USA and Uganda Bureau of Statistics. Interviews obtained data on respondent’s self-reported male circumcision status, sexual behaviors and social-demographic characteristics (age, marital status, highest education level, survey region, ethnicity, residence, religion and the wealth status of the households). HIV status was tested from blood samples obtained during the interviews from consenting respondents. Data from individual interview and HIV-status were linked by unique identifiers for each individual respondent. Of the 9,524 men aged 15–59 years, a total of 7969 (84%) ever sexually active men were linked to their HIV-status.

Risky sexual behavior was defined and grouped into four categories; i) transactional sex (*payment or receipt of money/gift in exchange for sex*) in the preceding 12 months of the survey, ii) life-time number of sexual partners, with 1–3 as the referent and 4 or more partners, based on the median life-time partners, 4, iii) non-marital sexual relations (*include non-cohabiting partners*), and iv) non-use of condoms with the last non-marital partner in the last 12 months. The definition of “non marital sex” is based on the UAIS (2011) and includes all men whether married or not, who were sexually active in the 12 months preceding the survey reporting having sex with a non-marital non cohabiting partner. “Condom use at last non marital sex” only included men who reported that they had non-marital sex in the preceding 12 months.

The outcome variables were the four RSBs, all coded either as ‘0’: when the behavior was not reported and ‘1’: when the behavior was reported, and for life-time partners coded as ‘0’: if reported as 1–3 lifetime partners and ‘1’: if 4+ partners were reported. The primary independent variable was self-reported male circumcision status, and social-demographic characteristics as other explanatory variables.

### Statistical analysis

Exploratory data analysis was conducted on all the variables of interest. In the bivariate analysis, cross tabulations were done to determine unadjusted associations between the outcomes (RSBs and HIV status), and circumcision status, and other covariates including social-demographic characteristics. The statistical significance was determined using chi-square tests at the 5% level. Odds ratios (OR) were used as the measure of association with their 95% confidence intervals (CI). These were obtained through logistic regression models. For the adjusted analyses, all variables in the bivariate analysis that had p < 0.15, OR >2 or OR < 0.5 or known confounders in the association were included in the multivariable logistic regression model. Collinearity of independent variables in the model was assessed by use of the variance inflation factor (VIF) and tolerance in STATA, where only non-collinear variables were retained. All the models had circumcision status as the primary independent factor.

Social-demographic characteristics included in these analyses were residence, marital status, religion, education, wealth status, region, age, and ethnicity.

The analysis was weighted in order to account for the complex survey methodology using the ‘svyset’ command in STATA. The AIS was reviewed and approved by the Science and Ethics Committee of the Uganda Virus Research Institute, ICF International’s Institutional Review Board, and a review committee at the Centers for Disease Control and Prevention in Atlanta. It was also cleared by the Ethics Committee of the Uganda National Council of Science and Technology. Permission to use this data was obtained from ICF international, USA.

## Results

### Description of the respondents

Table 1 shows respondents’ characteristics. Nearly 4 in 5 (81%) of the respondents were from rural areas, just over a half (57%) had primary education, nearly three quarters (72%) were either married or cohabiting, and almost a third (31%) were aged 25–34 years. The largest tribal grouping were Baganda (17%), and 13% of Muslim religion. Twenty eight percent (46% Muslim, 54% non Muslim) of the respondents reported that they were circumcised.

**Table 1:** Respondents’ characteristics and HIV status

	**All men**	**Circumcised**	**Uncircumcised**	**p - value**
	**n (%)**	**n (%)**	**n (%)**	
Overall	7969 (100)	2228 (100)	5741 (100)	
***Characteristics***				
**Age group**				
15-24	1941 (24.3)	610 (27.4)	1331 (23.2)	0.046
25-34	2460 (30.9)	708 (31.8)	1751(30.5)	0.527
35-44	2000 (25.1)	508 (22.8)	1492 (26.0)	0.151
45-59	1568 (19.7)	402 (18.0)	1166 (20.3)	0.317
**Residence**				
Urban	1520(19.1)	604 (27.1)	916 (16.0)	<0.001
Rural	6449 (80.9)	1624 (72.9)	4825 (84.0)	<0.001
Survey region				
Central	1784 (22.4)	491 (22.0)	1293 (22.5)	0.821
Kampala	568 (7.1)	215 (9.6)	353 (6.2)	0.135
Eastern	1701 (21.3)	882 (39.6)	819 (14.3)	<0.001
Northern	1999 (25.1)	201 (9.0)	1798 (31.3)	<0.001
Western	1916 (24.0)	439 (19.7)	1477 (25.7)	0.010
**Highest education level**				
No education	570 (7.2)	143 (6.4)	427 (7.4)	0.688
Primary	4526 (56.8)	1166 (52.3)	3360 (58.5)	<0.001
Secondary	2155 (27.0)	697 (31.3)	1458 (25.4)	0.004
Post secondary	718 (9.0)	222 (10.0)	496 (8.6)	0.545
**Marital status**				
Never married	1649 (20.7)	523 (23.5)	1127 (19.6)	0.070
Currently married	5710 (71.7)	1534 (68.9)	4176 (72.7)	0.005
Divorced/seperated	609 (7.6)	171 (7.7)	438 (7.6)	0.967
**Ethnicity**				
Baganda	1321 (16.6)	400 (18.0)	921 (16.1)	0.395
Banyakore	794 (10.0)	109 (4.9)	685 (11.9)	0.030
Iteso/Karimojong	730 (9.2)	64 (2.9)	667 (11.6)	0.032
Lugbara/Madi/Alur/Japadhola	783 (9.8)	186 (8.4)	597 (10.4)	0.426
Basoga	716 (9.0)	314 (14.1)	401 (7.0)	0.002
Langi/Acholi	896 (11.2)	19 (0.9)	877 (15.3)	0.082
Bakiga	427 (5.4)	42 (1.9)	385 (6.7)	0.222
Bagisu/Sabiny/	510 (6.4)	480 (21.6)	30 (0.5)	0.005
Bakonjo	170 (6.2)	165 (7.4)	5 (0.1)	0.533
Banyoro/Batoro	680 (8.5)	164 (7.4)	516 (9.0)	0.525
Bafumbira	165 (2.1)	24 (1.1)	141 (2.5)	0.672
Bagwere/Samia	280 (3.5)	99 (4.4)	181 (3.2)	0.607
Others	497 (6.2)	163 (7.3)	335 (5.8)	0.518
**Religion**				
Moslem	1038 (13.0)	1026 (46.1)	12 (0.2)	<0.001
Non Moslem	6931 (87.0)	1202 (53.9)	5729 (99.8)	<0.001
**HIV Status**				
Negative	7416 (93.1)	2120 (95.2)	5296 (92.3)	<0.001
Positive	553 (6.9)	108 (4.8)	445 (7.8)	0.280
**Circumcision status**				
Yes	2228 (27.9)			
No	5741 (72.0)			

In comparison to the uncircumcised, the circumcised men tended to be younger (age 15–24), from the urban areas, attained secondary education, Muslims, from eastern Uganda and of Bagisu, Sabiny and Basoga ethnicities, but did not differ by never married marital status.

HIV prevalence was 6.9% among all the men in the sample and the circumcised men were less likely to be HIV positive.

### Comparison of sexual behavior between circumcised and uncircumcised Men

Table 2 shows the comparison of the prevalence of RSBs among circumcised and uncircumcised men. Overall, nearly a third (32.5%) of the men reported non-marital sex in the last 12 months, and just over a half (55%) reported non-use of condoms the last time they had such sex. The least reported RSB was transactional sex (3%). All the four RSBs were most commonly reported among the circumcised men; nearly two thirds of circumcised compared to 56% of the uncircumcised men reported 4 or more life-time partners (p-value <0.001), while 38% of the circumcised compared to 30% of the uncircumcised reported non-marital sex (p-value <0.001).

**Table 2:** Comparison of risky sexual behaviors between circumcised and uncircumcised men

	**Circumcised**	**Uncircumcised**	**Overall**
	**n (%)**	**n (%)**	**n (%)**
**Risky sexual behavior**			
Non-marital partners	766 (38.4)	1547 (30.2)	2314 (32.5)
Non-use of condoms	319 (58.4)	728 (52.9)	1267 (54.7)
Transactional sex	74 (3.7)	139 (2.7)	214 (3.0)
4+ lifetime partners	1466 (65.8)	3239 (56.4)	4706 (59.1)

### Associations between circumcision status and sexual behaviors

Table 3 shows both unadjusted and adjusted associations between the different RSBs and circumcision status. Four models were run using each RSB as an outcome, with circumcision status as the primary independent variable. In the adjusted analysis we controlled for age, ethnicity, residence, wealth status, marital status, and education as covariates. In the final models, the variable region was dropped because it was found to be collinear with ethnicity, which was retained as a more important variable in explaining some of the key findings.

**Table 3:** Unadjusted and Adjusted Odds ratios for risky sexual behaviors comparing circumcised and uncircumcised

	**(1)**	**(2)**	**(3)**	**(4)**
	**Number of lifetime partners**	**Had non marital sex the last 12 months**	**Condom use at last non marital sex**	**Transactional sex in last 12 months**
	**ORs (95% CI)**	**ORs (95% CI)**	**ORs (95% CI)**	**ORs (95% CI)**
**Circumcision status**				
***Unadjusted***				
Uncircumcised	1	1	1	1
Circumcised	1.48^*?*^ (1.31,1.68)	1.43^*^ (1.24,1.65)	0.80^*^ (0.65,0.98)	1.37 (0.98,1.94)
***Adjusted***				
Uncircumcised	1	1	1	1
Circumcised	1.46^?^ (1.27,1.67)	1.26^*^ (1.05,1.52)	0.79^*^ (0.63,0.98)	1.20 (0.81,1.78)
***Background characteristics***^¶^				
**Age**				
15-24	1	1	1	1
25-34	1.81^?^ (1.52,2.16)	0.74^*^ (0.57,0.95)	0.86 (0.64,1.14)	0.71 (0.42,1.18)
35-44	3.12^?^ (2.56,3.79)	0.61^?^ (0.46,0.78)	0.66^*^(0.44,0.96)	0.66 (0.37,1.16)
45-59	4.26^?^ (3.44,5.28)	0.36^?^ (0.27,0.48)	0.34^?^ (0.22,0.53)	0.22^?^(0.11,0.44)
**Ethnicity**				
Baganda	1	1	1	1
Banyakore	0.54^?^ (0.41,0.72)	0.77 (0.51,1.14)	0.66 (0.60,1.08)	0.80 (0.30,2.12)
Iteso/Karimojon	1.83 (0.64,1.08)	0.51^?^ (0.34,0.75)	0.57^*^ (0.37,0.89)	0.56 (0.16,1.89)
Lugbara/Madi/Alur/Japadhola	0.82 (0.62,1.09)	0.62^?^ (0.44,0.88)	0.92 (0.87,2.45)	1.79 (0.81,3.95)
Basoga	1.32^*^ (1.03,1.69)	1.17 (0.73,1.86)	0.46^?^ (0.30,0.70)	1.34 (0.44,4.02)
Langi/Acholi	0.88 (0.67,1.15)	0.53^?^ (0.37,0.75)	0.79 (0.51,1.20)	0.20 (0.05,0.78)
Bakiga	0.67^*^ (0.49,0.92)	0.67 (0.40,1.11)	0.30^?^ (0.19,0.46)	2.05^?^ (1.03,4.10)
Bagisu/Sabiny	1.06 (0.76,1.66)	0.58^*^ (0.34,0.98)	0.40^?^ (0.25,0.63)	0.99 (0.37,2.65)
Banyoro/Batoro	1.41^*^ (1.06,1.88)	1.61^*^ (1.12,2.32)	0.50^?^ (0.33,0.76)	2.47^*^(1.21,5.07)
Bafumbira	0.32^?^ (0.18,0.55)	0.43^*^ (0.20,0.89)	0.25^?^ (0.09,0.71)	0.27 (0.03,2.47)
Bagwere/Samia	1.19 (0.79,1.79)	1.17 (0.72,1.90)	0.78 (0.41,1.52)	1.50 (0.57,3.94)
Bakonjo	0.61^*^ (0.38,0.98)	0.83 (0.53,1.31)	0.23^*^ (0.07,0.72)	0.79 (0.18,3.37)
Others	0.86 (0.63,1.18)	0.95 (0.62,1.45)	0.96 (0.63,1.46)	1.72 (0.88,3.35)
**Wealth status**				
Lowest quintile	1	1	1	1
2nd quintile	1.30^?^ (1.09,1.53)	1.31^*^ (1.00,1.72)	1.12 (0.76,1.65)	0.92 (0.46,1.85)
Middle	1.48^?^ (1.22,180)	1.59^?^ (1.20,2.09)	1.40 (0.95,2.06)	1.77 (0.87,3.59)
4th quintile	1.61^?^ (1.34,1.93)	2.02^?^ (1.50,2.71)	1.75^?^ (1.17,2.62)	1.28 (0.62,2.67)
Highest quintile	1.97^?^ (1.56,2.48)	1.98^?^ (1.37,2.85)	2.55^?^ (1.63,3.99)	1.11 (0.45,2.70)
**Marital status**				
Never married	1	1	1	1
Married	2.29^?^ (1.90,2.75)	0.01^?^ (0.00,0.01)	1.35^*^ (1.04,1.77)	0.72 (0.41,1.29)
Divorced/separated	2.55^?^ (1.98,3.27)	0.07^?^ (0.04,0.12)	1.13 (0.79,1.61)	2.21^*^(1.17,4.17)
**Highest education level**				
No education	1	1	1	1
Primary	1.46^?^ (1.17,1.83)	1.07 (0.78,1.46)	2.18^*^ (1.13,4.21)	0.92 (0.41,2.07)
Secondary	1.42^?^ (1.09,1.86)	1.06 (0.74,1.52)	3.14^?^ (1.63,6.04)	0.67 (0.24,1.86)
Post secondary	1.07 (0.77,1.48)	1.29 (0.86,1.96)	3.57^?^ (1.79,7.14)	0.42 (0.12,1.48)
**Number of men**	**7,969**	**7,114**	**2,231**	**7,109**

*p < 0.05,^*?*^*p < 0.01,*^*¶*^*Includes only background characteristics that were significant in at least one of the 4 models. Survey region is omitted because of collinearity. Only indiciating the background factors that were significant.*

Male circumcision was significantly associated with increased odds of having non-marital sex, and reporting 4+ lifetime sexual partners. However, non-use of condoms was significantly lower among the circumcised compared to the non- circumcised. In models 2 and 3, the odds of having non marital sex in the last 12 months were 1.26 times higher among the circumcised men compared with the uncircumcised while non use of condoms at the last such sex was also significantly associated with circumcision status (p < 0 .05).

### Associations between circumcision status and HIV status

Table 4 (Model 1) shows the adjusted association between HIV status and circumcision status, controlling for age, ethnicity, residence, wealth status, marital status, and highest level of education. Survey region was found to be collinear with ethnicity and thus dropped. In model two we controlled for both background characteristics as in model one, and two of the RSBs (number of lifetime sexual partners and transactional sex in the last 12 months). The other RSBs (non marital sex and condom use at last such sex were excluded because of collinearity). Results in model 1 indicate that the odds of being HIV positive among circumcised men were 35% lower compared with uncircumcised men after controlling for the five remaining background characteristics as indicated above, while in model 2, the odds of being HIV positive among circumcised men were 37% lower compared with uncircumcised men after controlling for both background characteristics and the two RSBs.

**Table 4:** **Adjusted Odds ratios for Circumcision status and HIV**^**+ **^**status**

	**(1)**	**(2)**
	**HIV status, adjusted for background characteristics**	**HIV status, adjusted for background characteristics and risky sexual behaviors**^¶^
	**ORs (95% CI)**	**ORs (95% CI)**
**Circumcision status**		
Uncircumcised	1	1
Circumcised	0.65^*^ (0.47,0.90)	0.63^*?*^ (0.45,0.87)
***Background characteristics***^¶^		
**Age**		
**Age group**	1	1
15-24	2.21^*?*^ (1.27,3.81)	2.08^*?*^ (1.21,3.57)
25-34	3.91^*?*^ (2.27,6.71)	3.49^*?*^ (2.05,5.93)
35-44	2.73^*?*^ (1.57,4.79)	2.38^*?*^ (1.37,4.12)
**Ethnicity**		
Baganda	1	1
Banyakore	1.42 (0.85,2.39)	1.50 (0.90,2.51)
Iteso/Karimojong	1.35 (0.77,2.39)	1.35 (0.75,2.42)
Lugbara/Madi/Alur/Japadhola	1.14 (0.59,2.22)	1.13 (0.58,2.21)
Basoga	1.24 (0.69,2.22)	1.20 (0.67,2.16)
Langi/Acholi	1.53 (0.80,2.95)	1.57 (0.81,3.06)
Bakiga	1.53 (0.86,2.73)	1.55 (0.88,2.73)
Bagisu/Sabiny	1.50 (0.77,2.92)	1.43 (0.74,2.77)
Banyoro/Batooro	1.51^*^ (1.00,2.27)	1.56 (0.99,2.47)
Bafumbira	0.95 (0.47,1.91)	1.15 (0.57,2.33)
Bagwere/Samia	2.10 (0.90,4.92)	1.98 (0.83,4.71)
Bakonjo	0.52 (0.17,1.62)	0.57 (0.19,1.76)
Others	1.58 (0.97,2.59)	1.69^*^ (1.01,2.85)
**Marital status**		
Never married	1	1
Currently married	1.69 (0.97,2.92)	1.62 (0.94,2.82)
Divorced/separated	4.13^*?*^ (2.34,7.28)	3.65^*?*^ (2.10,6.32)
**Highest education level**		
No education		
Primary	1.11 (0.72,1.71)	1.07 (0.71,1.64)
Secondary	0.95 (0.58,1.56)	0.95 (0.59,1.53)
Post secondary	0.56^*^ (0.32,0.98)	0.59 (0.34,1.02)
**Wealth Status**		
Lowest quintile	1	1
2nd quintile	1.10 [0.79,1.52]	1.06 (0.77,1.47)
Middle	1.15 (0.79,1.69)	1.10 (0.75,1.61)
4th quintile	1.52* (1.04,2.22)	1.47 (1.00,2.18)
Highest quintile	1.39 (0.89,2.17)	1.52* (1.02,2.27)
**Transactional sex in last 12 months**		
Did not pay for sex		1
Paid for sex		2.17^*?*^ (1.29-3.66)
**Number of lifetime partners**		
Less than four		1
Four or more		1.75^*?*^ (1.40-2.20)
**Number of men**	**7,969**	**7,969**

^*^p < 0.05,^*?*^*p < 0.01,*^¶^*Non marital sex and condom use at non marital sex and survey region were omitted because of collinearity.*

## Discussion

The findings from our study indicate that over a quarter of men who were ever sexually active were circumcised and one third of these were aged between 25 to 34 years. The most common risky sexual behaviors were multiple life-time sexual partners (4+), having non marital sex, and non-use of condoms during non marital sex. These were also significantly higher among the circumcised. Despite evidence of higher RSB among the circumcised men, we found lower HIV prevalence in this group compared to their uncircumcised counterparts.

Circumcised men had higher odds of having 4 or more life time sexual partners, engaging in non marital sex in the 12 months preceding the survey and non-condom use at last such sex than uncircumcised men. In a Zimbabwe study [20], circumcision was not associated with RSBs, but instead uncircumcised men who were willing to be circumcised had more risky behaviors, while in a post trial study in Rakai-Uganda [21], there was no evidence of risk compensation as well. However, our results are consistent with findings from another study [22], which showed that circumcised men had unprotected sexual intercourse and more sexual partners. Some studies attribute such unexpected differences in sexual behavior to the behavior risk compensation where men change their sexual behaviors with the knowledge that their risk of infection is reduced [17,19,23]. In the study by Riess et al. (2010), some men stopped using condoms temporarily after undergoing male circumcision as part of the new program in Kisumu (Kenya) while others increased the number of sexual partners, but the study overall reported no sexual behavioral disinhibition. In the South African RCT, circumcised men reported more sexual contacts than uncircumcised men at the 4– 12 month and in the 13 to 21 month recall periods [5]. The promotion of SMC without increased education and counseling of the men may hinder progress in further HIV reduction [24] as circumcised men had more RSBs. Even though there is no empirical evidence in this study to ascertain that the RSBs observed among the circumcised men were a result of risk compensation, it may be one of the possible explanations. Another possibility could be that men that already have RSBs decide to undergo circumcision to reduce their chances of HIV infection. These may not change behaviors post circumcision, but this needs more exploration.

In relation to circumcision and HIV status, results at both bivariate and multivariate levels showed that circumcised men were more likely to be HIV negative compared with the uncircumcised. This protective effect of circumcision is consistent with results from the 3 RCTs [3-5] that showed the same protection against heterosexual HIV transmission from infected women to men. More importantly in our study, this protective effect was observed even though the odds of RSBs were much higher among the circumcised men. This could mean that the negative effect of risk compensation in our context may be insignificant compared to the fundamental benefits of the SMC interventions in this population.

The limitations to this study are that: analysis is based on data from a cross-sectional survey design and thus not possible to establish causality using such data, the analysis was restricted to only men who were ever sexually active, while the circumcision status and sexual behaviours were self reported by participants.

## Conclusions

Circumcision was strongly associated with RSB. However, HIV prevalence was significantly lower in circumcised men.

Safe male circumcision messages need to continue to emphasise the risk of HIV even after circumcision. Intensified individual tailored counseling pre and post SMC procedures may play a role in reducing these behaviors.

More sensitizations both at population level and at health facilities on the advantages of circumcision need to be done so as to encourage more men to get circumcised given the protective effect observed even amidst RSBs.

## Abbreviations

ABC: Abstinence, being faithful and condom use; RCTs: Randomised controlled trials; AIDS: Acquired immune deficiency syndromme; AIS: AIDS indicator survey; HIV: Human immune virus; MOH: Ministry of health; RSB: Risky sexual behaviour; SMC: Safe male circumcision; STIs: Sexually transmitted infections.

## Competing interests

The Authors declare that they have no competing interest.

## Authors’ contribution

SPSK conceived the study, led the data analysis and writing process. EN substantially contributed to conceiving the study, participated in data analysis and writing. NMT participated in the analysis and writing. LMA contributed to the writing. FM contributed significantly to the analysis and writing. All authors read and approved the final manuscript.

## Authors’ informations

SPSK is a social and population scientist and an assistant lecturer at the school of public health, Makerere University.

EN is a population scientist and assistant lecturer at the school of statistics and planning, Makerere University.

NMT is a biostatistician and associate professor of epidemiology at the school of public health, Makerere University.

LMA is a social scientist and lecturer at the school of public health, Makerere University.

FM is a biostatistician and senior lecturer at the school of public health, Makerere University.
